# Characteristic SNPs defining the major multidrug-resistant *Mycobacterium tuberculosis* clusters identified by EuSeqMyTB to support routine surveillance, EU/EEA, 2017 to 2019

**DOI:** 10.2807/1560-7917.ES.2024.29.12.2300583

**Published:** 2024-03-21

**Authors:** Albert J de Neeling, Elisa Tagliani, Csaba Ködmön, Marieke J van der Werf, Dick van Soolingen, Daniela Maria Cirillo, Richard M Anthony

**Affiliations:** 1 National Tuberculosis Reference Laboratory, Centre for Infectious Disease Control, National Institute for Public Health and the Environment (RIVM), Bilthoven, the Netherlands; 2 Emerging Bacterial Pathogens Unit, Division of Immunology, Transplantation and Infectious Diseases, IRCCS San Raffaele Scientific Institute, Milan, Italy; 3 European Centre for Disease Prevention and Control (ECDC), Stockholm, Sweden

**Keywords:** Mycobacterium, tuberculosis, cluster, SNP, MDR-TB

## Abstract

**Background:**

The EUSeqMyTB project, conducted in 2020, used whole genome sequencing (WGS) for surveillance of drug-resistant *Mycobacterium tuberculosis* in the European Union/European Economic Area (EU/EEA) and identified 56 internationally clustered multidrug-resistant (MDR) tuberculosis (TB) clones.

**Aim:**

We aimed to define and establish a rapid and computationally simple screening method to identify probable members of the main cross-border MDR-TB clusters in WGS data to facilitate their identification and track their future spread.

**Methods:**

We screened 34 of the larger cross-border clusters identified in the EuSeqMyTB pilot study (2017–19) for characteristic single nucleotide polymorphism (SNP) signatures that could identify and define members of each cluster. We also linked this analysis with published clusters identified in previous studies and identified more distant genetic relationships between some of the current clusters.

**Results:**

A panel of 30 characteristic SNPs is presented that can be used as an initial (routine) screen for members of each cluster. For four of the clusters, no unique defining SNP could be identified; three of these are closely related (within approximately 20 SNPs) to one or more other clusters and likely represent a single established MDR-TB clade composed of multiple recent subclusters derived from the previously described ECDC0002 cluster.

**Conclusion:**

The identified SNP signatures can be integrated into routine pipelines and contribute to the more effective monitoring, rapid and widespread screening for TB. This SNP panel will also support accurate communication between laboratories about previously identified internationally transmitted MDR-TB genotypes.

**Key public health message**

**What did you want to address in this study and why?**

*Mycobacterium tuberculosis* causes tuberculosis (TB). Whole genome sequencing (WGS) is a technique increasingly applied to identify and type *M. tuberculosis* in Europe. Its high accuracy allows probable infections between patients, and clusters of TB to be detected, supporting infection control efforts. WGS is rapid and effective within a single laboratory but communication about linked isolates between laboratories could be improved.

**What have we learnt from this study?**

In this study, we identify a series of genetic markers that can be used to simply and rapidly screen for drug-resistant clones of *M. tuberculosis* that belong to previously identified cross-border transmission clusters of multidrug-resistant TB in the EU/EEA, supporting fundamental research on the epidemiology of this disease.

**What are the implications of your findings for public health?**

The availability of these genetic markers will allow laboratories generating genome sequences from *M. tuberculosis* isolates to rapidly screen their data to determine if any of their isolates are potentially members of previously identified drug-resistant clusters and improve communication between laboratories.

## Introduction

Rifampicin resistant/multidrug-resistant *Mycobacterium tuberculosis* complex (RR/MDR-MTBC) infections are difficult to treat, requiring expensive drugs for an extended period. The spread of pre-existing MDR-TB clusters contributes significantly to the burden of MDR-MTBC in the EU [1]. In 2020, the ‘Pilot study on the use of whole genome sequencing for molecular typing and characterisation of *M. tuberculosis* in the EU/EEA**’** (EuSeqMyTB project) assembled and analysed in detail sequence data from 2,218 RR/MDR-MTBC isolates collected in 25 European Union/European Economic Area (EU/EEA) countries [1]. This sample represented over 75% of all multidrug-resistant (MDR) tuberculosis (TB) cases reported in the region between January 2017 and December 2019. Thus, this dataset provides a valuable insight into the genetic structure of MDR-TB isolates in the EU/EEA during the study period, as well as a resource to monitor the future evolution of MDR-TB in the region. This type of study is essential to trace possible active transmission across country borders and to facilitate more fundamental research on the epidemiology. Furthermore, it supports the identification of factors underlying successful international spread of MDR-TB. Genotyping of MDR-TB isolates in the EUSeqMyTB project was achieved by assembling raw sequencing data in a single location along with basic clinical information from cases, and then analysing the data using the MTBseq pipeline [2]. The main international MDR-TB clusters uncovered in this project were identified on the basis of single nucleotide polymorphism (SNP) distance as described in [1], and included a total of 56 international clusters, 34 of which containing three or more isolates from at least two countries.

At present, the available description of these clusters does not allow newly sequenced isolates to be easily associated with the MDR-TB clusters identified in local settings, unless FASTQ files are exchanged and analysed in the same dataset using a common analytical pipeline [3-5]. Also, as the clusters were identified on the basis of SNP distances between strains within the dataset, the structure and membership of a cluster can vary depending on the specific strains present in the database. For example, if a strain is added that falls within the cluster SNP threshold of two clusters that differ from each other by slightly more than the cluster threshold selected upon reanalysis, these clusters will merge and become a single cluster. Real-time monitoring for cross-border well-defined MDR-TB clades should ideally be integrated into local pipelines and be simply performed within local workflows with very low computational burden [6]. 

A list of 62 ‘Coll SNPs’ [7] that can be used to accurately and simply identify the main MTBC lineages is already implemented in many EU/EEA MTBC bioinformatic pipelines. Here, we explore the possibility of generating a panel of SNPs to allow the simple identification of cross-border MDR-TB clusters described in the EUSeqMyTB pilot study (termed snpCLs). Such a SNP panel could be used to perform an initial screen for members of these genetic clades in the same way as the Coll SNPs.

There have been previous initiatives to describe clustered MDR-TB isolates within the EU [8]. Linking to these previously described cluster datasets and defining clusters in a way that allows them to be linked with future clusters identified is also desirable, and the availability of clade defining SNPs will aid the tracking of these clusters over time. In an earlier survey, a very large MIRU-VNTR MDR-TB cluster designated ECDC0002 present in the EU/EEA was described and some members of the cluster subjected to WGS [8]. Based on this work a *rpoC* mutation was identified that was at that time uniquely associated with this cluster [9]. As a proof of concept to assess the validity of our approach, we also screened the EUSeqMyTB database for this previously reported characteristic SNP.

## Methods

### Dataset analysed

We analysed all 34 cross-border MDR-TB clusters of three or more isolates described by Tagliani et al. [1] to identify cluster-characteristic SNPs. A minimum cluster size of three isolates was used, as we consider these clusters the most interesting simply because they were the larger clusters (> 2 isolates) and present in more than one country. 

### Analysis approach

The complete SNP SQL database, based on mapping unpaired Illumina (Illumina Inc.) reads to the H37Rv reference genome version 3.0 (GenBank accession number AL123456.3) consisting of SNPs detected using Bowtie2 in Breseq version 0.28.1 [10] using standard settings, i.e. a minimum allele frequency of 80% and a minimum coverage of five reads, was screened for SNPs at any position in the genome without excluding any gene. SNPs were identified for each cluster (snpCL) and univocally assigned to each cluster only if present in all its members and absent in all other isolates in the EUSeqMyTB database. These SNPs were termed ‘cluster-specific SNPs’. If a unique cluster-specific SNP could not be identified, the clusters were expanded by sequentially adding the genetically closest isolate until a unique SNP could be identified. If a characteristic SNP was not found before the snpCL cluster merged with another snpCL cluster, then no unique SNP was defined for that cluster.

### Identifying isolates linked to the largest previously identified EU/EEA cluster

The database was also screened for the previously described ECDC0002 cluster [9], by identifying all isolates carrying the previously reported characteristic SNP (764724_C in the *rpoC* gene).

## Results

### Cluster-defining SNPs identified

For 30 of the 34 published snpCLs comprising three or more isolates from two or more countries, it was possible to identify a unique SNP suitable for screening of these clusters (Table 1). Seven of these clusters had to be expanded by including additional isolates, up to a maximum of five per cluster, to identify a characteristic SNP. All these additional isolates (n = 17) were within 20 base pairs (bp) of their respective snpCL (https://github.com/KeesJohannes/spanningTree).

**Table 1:** Proposed clade-defining single nucleotide polymorphisms for the previously reported EUSeqMyTB snpCL[1], EU/EEA, 2017–2019 (n = 34 clades)

SNP	snpCL^a^	Gene product	Locus tag	Amino acid change (codon)^b^	Size of snpCL	Isolates containing the proposed SNP^c^	Genotype (Coll clade)
1157317_T	1	Two component sensor histidine kinase TrcS	Rv1032c	Leu213Leu (ctg/ctA)	29	32	Mainly T (4.8)
4327088_C	2	Monooxygenase EthA	Rv3854c	Leu129Arg (ctc/cGc)	20	20	Ural (4.2.1)
130881_G	3	NA			16	16	Euro-American (4.6.2)
1485300_G	4	NA			14	15	Mainly T (4.8)
1208477_G	5	Hypothetical protein	Rv1084	Ala281Gly (gcg/gGg)	13	15	Ural (4.2.1)
1547828_C	6	NA			13	14	Mainly T (4.8)
2971513_G	7	Integrase	Rv2646	Asp321Glu (gac/gaG)	12	17	Beijing (2.2.1)
1039921_G	8	NA			12	12	Euro-American (4.2.2)
No SNP	9	NA			12	NA	Mainly T (4.8)
211015_A	10	Transmembrane protein	Rv0180c	His412His (cac/caT)	12	12	Haarlem (4.1.2.1)
1895566_G	11	NA			10	10	LAM (4.3.3)
1914071_A	12	Tyrosine-tRNA ligase	Rv1689	Arg157Gln (cgg/cAg)	10	10	Euro-American (4.2.2)
1810244_A	13	Indole-3-glycerol phosphate synthase	Rv1611	Ser2Asn (agt/aAt)	9	9	Beijing (2.2.1)
4155977_A	14	DNA polymerase III subunit epsilon	Rv3711c	Val251Val (gtc/gtT)	7	7	Beijing (2.2.1)
2399093_A	15	Dihydroorotate dehydrogenase	Rv2139	Arg125Gln (cgg/cAg)	7	7	Beijing (2.2.1)
1008074_A	16	Acetyl-CoA carboxylase carboxyl transferase beta	Rv0904c	Ala36Val (gcg/gTg)	7	8	Beijing (2.2.1)
4284172_T	17	Hypothetical protein	Rv3819	Asp59Asp (gac/gaT)	5	9	LAM (4.3.3)
766707_G	18	DNA-directed RNA polymerase subunit beta	Rv0668	Glu1113Gly (gaa/gGa)	5	5	Beijing (2.2.1)
3169491_A	19	Aldehyde dehydrogenase	Rv2858c	Ser411Ser (tcg/tcT)	5	5	Mainly T (4.8)
996197_C	20	S-adenosylmethionine-dependent methyltransferase	Rv0893c	Asp33Glu (gat/gaG)	5	5	LAM (4.3.3)
No SNP	21	NA			5	NA	TUR (4.2.2.1)
1231934_G	22	NA			5	6	Haarlem (4.1.2.1)
1398622_A	23	Hypothetical protein	Rv1251c	Arg207Cys (cgc/Tgc)	4	4	LAM (4.3.3)
No SNP	24	NA			4	NA	Beijing (2.2.1)
1068731_C	25	Formyltransferase/inosine monophosphate cyclohydrolase	Rv0957	Arg176Thr (agg/aCg)	3	3	Beijing (2.2.1)
No SNP	26	NA			3	NA	Beijing (2.2.1)
3031515_C	27	Membrane protein	Rv2719c	His8Arg (cat/cGt)	3	3	Beijing (2.2.1)
1028437_T	28	Transposase	Rv0922	Arg251Arg (cgc/cgT)	3	3	Mainly T (4.7)
3640351_T	29	NA			3	3	Mainly T (4.8)
3223901_A	30	NA			3	3	Mainly T (4.8)
1946519_T	31	NA			3	3	Euro-American (4.2.2)
3088899_A	32	Oxidoreductase	Rv2781c	Ala29Val (gcg/gTg)	3	3	X-type (4.1.1.1)
1921877_A	33	Hypothetical protein	Rv1697	Gly112Gly (ggg/ggA)	3	3	Haarlem (4.1.2.1)
1760095_G	34	Fumarate reductase iron-sulphur subunit	Rv1553	Pro221Ala (cct/Gct)	3	3	Haarlem (4.1.2.1)

EU/EEA: European Union/European Economic Area; NA: not applicable; SNP: single nucleotide polymorphism.

^a^ The snpCL1 was expanded by 3 isolates to identify a characteristic SNP and one isolate was missed because of a mixed genotype. SNP 3640351 c > T, which defines snpCL29, is within 4 bp of a second SNP 3640354 c > G.

^b^ A capital letter is used to indicate the changed base.

^c^ The total number of isolates from the 2,218 isolates in the EUSeqMyTB database containing the proposed SNP.

For four SNP clusters (snpCL9, snpCL21, snpCL24 and snpCL26), no characteristic unique SNP was identified that fulfilled the pre-assigned criteria. Additional analysis showed that three of these clusters were within 20 SNPs to one or more other clusters: snpCl9 to snpCL1 and snpCl24, and snpCl26 to snpCl16.

### Isolates related to the ECDC0002 cluster 

The EUSeqMyTB database contained a total of 107 isolates with a mutation in position 764742 in the *rpo*C codon 452. Of those 107 isolates, 99 had the previously reported Phe452Ser (ttc/tCc) mutation [9], and the remaining eight had a different mutation (Phe452Cys (ttc/tGc). Of the eight isolates with a Phe452Cys (ttc/tGc) mutation, six were Beijing (2.2.1), one was Delhi CAS (3.1.1) and one mainly T (4.7). Most isolates (97/99) with the t > C mutation were Beijing (2.2.1) (Table 2), and all 97 Beijing (2.2.1) isolates were within 70 SNPs distance of each other (https://github.com/KeesJohannes/spanningTree). However, the remaining two isolates carrying this mutation belonged to a different lineage (LAM 4.3), demonstrating that this mutation was not fully specific for these related isolates and is likely a compensatory mutation associated with rifampicin resistance. Thus, an alternative SNP to uniquely define these 97 isolates, the Arg179Cys (cgc/Tgc) mutation in the *echA*11 gene (position 1268475 C > T, enoyl-CoA hydratase *echA*11, Rv1141c), was identified. This mutation was specific for and perfectly defined the 97 isolates related to the ECDC0002 cluster (Table 2). All members of the cross-border clusters snpCL16 (n = 7), snpCL24 (n = 4) and snpCL26 (n = 3) carried this mutation and were sub-clusters of the ECDC0002 [8] cluster.

**Table 2:** Distribution of the *rpo*C 452 and *ech*A11 179 mutation in the EUSeqMyTB database (n = 2,218 isolates) and correlation with the previously described ECDC0002 *rpo*C cluster (n = 452), EU/EEA, 2018 [9]

SNP relative to the reference genome at position 764724	Number of isolates in the EUSeqMyTB database	Number of isolates per Genotype (Coll clade)	Number of isolates also with the c > T 1268475 *echA*11 Arg179Cys SNP
t > C SNP at 764724 in *rpo*C Phe452Ser (ttc/tCc)	99	97 Beijing (2.2.1)	97 (includes snpCL16, snpCL24 and snpCL26)
		2 LAM (4.3)	0
t > G SNP at 764724 in *rpo*C Phe452Cys (ttc/tGc)	8	6 Beijing (2.2.1)	0
		1 Delhi CAS (3.1.1)	0
		1 mainly T (4.7)	0
Wild type 764724 *rpo*C 452 (ttc)	2,111	Various	0

EU/EEA: European Union/European Economic Area; SNP: single nucleotide polymorphism.

## Discussion

Here we describe a panel of SNPs to screen for members of cross-border clusters of MDR-TB identified in the EUSeqMyTB project [1]. Using this approach, we were able to univocally identify by a unique SNP variant all the members belonging to 30 of 34 previously described European cross-border clusters [1]. For only one cluster, no SNP signature could be univocally defined, while for the remaining three clusters a unique SNP could be found after incrementally increasing the cluster threshold up to a 20 SNP distance. These SNPs can be used in the same way as the Coll SNPs [7] to preliminarily identify specific clades, allowing simple integration into local pipelines. Additionally, this panel of SNPs will allow future clusters to be easily linked back to these clades, as demonstrated here with three snpCLs (snpCL16, snpCL24 and snpCL26) which, on the basis of previously published characteristic SNPs, belong to the B0/W148 [11] genotype and are sub-clusters of the previously identified European ECDC0002 MDR-TB cluster [8,9].

As we looked for mutations uniquely present in the clusters of interest, it was not necessary to eliminate poorly mapped regions. The possibility that the identified variants are the results of analytical errors can effectively be excluded, as the selected SNPs were uniquely present in only very closely clustered isolates and absent in all other isolates in the database. Poorly mapped and unreliably called SNPs would be expected to be miss-called in completely unrelated strains at least once in the over 2,000 records present in the screened database. Notably, two of the SNPs selected are not included in our standard SNP distance calculations. For snpCL28, the variant in position 1028437 c > T which defines snpCL28 is located in a transposase gene (Rv0922), and for snpCL29, the variant in position 3640351 c > T would be excluded in most SNP routine calling algorithms [12]. Other researchers have also observed that reliable calling of SNPs in these generally excluded regions is possible [13,14]. Importantly, nine of the 30 characteristic SNPs identified occur outside annotated reading frames and thus would not be captured using most core genome multi locus sequence typing (MLST) typing systems. 

The use of characteristic SNPs in a clonal organism also provides the possibility to effectively screen for mixed infections. Screening the entire genome for mixed loci is at present complex to routinely implement but screening a short list of genotypically informative SNPs can be easily realised. For example, our pipeline routinely screens the purity of Coll SNPs to check for mixed genotypes and the ribosomal genes for mixed species, although the exact threshold of reads needed to make a confident call is yet to be defined [5].

Linking to previous datasets is desirable as it allows transmission of successful clones to be monitored. In an earlier survey of MDR-TB in the EU/EEA region supported by the European Centre for Disease Prevention and Control (ECDC) and based on MIRU-VNTR typing [8], a very large MIRU-VNTR MDR-TB cluster designated ECDC0002 was described. This cluster was found to be identical to a previously observed dominant cluster EU0051 [15] and was also shown to be closely related to the Europe–Russia B0/W148 outbreak previously described [16-18] and Beijing lineage strain MtbC 15–9 type 100–32 [19]. The ECDC0002 cluster consisted of 452 *M. tuberculosis* isolates with identical MIRU-VNTR profiles, consistent with the Beijing lineage, all of which had either an MDR-TB or (pre-) extremely drug resistant( XDR)-TB profile [9]. All members of this cluster carried a specific mutation in the *rpo*C gene (F452S, T764724C) which, at the time of the previous ECDC surveillance study, was unique to this genotype. In the EUSeqMyTB database, mutations at this position of the *rpo*C gene are present in a number of unrelated isolates, supporting an adaptive role for this mutation with respect to rifampicin resistance [20], as was already suspected in the initial report [9]. For this reason, a second SNP was identified in the *ech*A11 gene (Arg179Cys (cgc/Tgc)). This SNP is unique for 97 Beijing 2.2.1 isolates, which are all within a 45 SNPs distance from each other, suggesting that this clone has been circulating in Europe for decades. As the 2003–11 ECDC TB surveillance study [8] relied on VNTR typing, it is not known whether this clone was the result of recent transmission at that time, or it was an already disseminated MDR-TB clone. Nonetheless, 14 of these 97 isolates were members of snpCL16, snpCL24 or snpCL26 [1], suggesting ongoing transmission of daughter clusters of this MDR clade in Europe.

For four snpCLs (snpCL 9, 21, 24 and 26), a SNP signature could only be found upon increasing the cluster threshold from 6 to 20 SNPs, which led to the merging of these clusters with other ‘related’ clusters, e.g. snpCL9 with snpCL1, suggesting that these snpCL clusters represent sub-clusters of established clades possibly combined with under-sampling, i.e. missing isolates that may have linked closely related clusters within the SNP thresholds chosen.

If the clustered clones identified in the EUSeqMyTB project continue to expand, the SNPs presented here will allow them to be tracked even if this is the result of a series of MDR-TB divergent sub-clusters. In time, cases involving these now established clones may no longer be the result of direct transmission, as is already the case for the previously identified ECDC0002 cluster. Patient interviews and detailed epidemiological investigations are needed to definitively establish transmission chains, but accurate rapid genetic screening helps to target epidemiological investigations [4]. 

We acknowledge some limitations. Our analysis was limited to SNPs. It is conceivable that characteristic insertions, deletions or genetic rearrangements were also present. Most of the current pipelines do not routinely utilise this variability to genotype *M. tuberculosis* isolates, but this may be possible in the future.

## Conclusion

The SNPs signatures described here and in similar studies can be integrated into routine *M. tuberculosis* WGS pipelines in the same way as the Coll SNPs, and can contribute to the more effective monitoring, rapid and widespread screening, as well as investigation and communication relating to these transmitted clones. With the recently established EpiPulse platform, more internationally clustered isolates will be identified and hopefully future curated panels of characteristic SNPs created in order to encompass emerging clones. Such lists would ideally be maintained by the ECDC or laboratory networks such as the European Reference Laboratory Network for TB (ERLTB-Net). Defining identified clusters with a SNP profile will facilitate accurate and hopefully more rapid communication to monitor their spread.
